# On the Theory of Simple Epilepsy

**Published:** 1861-09

**Authors:** Charles Bland Radcliffe

**Affiliations:** Physician to the Westminster Hospital


*On the Theory of Simple Epilepsy.* By Dr. Charles Bland Radcliffe, Physician to the Westminister Hospital.

In the epileptic fit itself, the facts, when fairly read, admit of one conclusion, and one only. At the instant of the fall, a corpse-like pallor overspreads the countenance and the pulse dies out at the wrist—phenomena which seem to be only intelligible on the supposition that the arteries are nearly empty of red blood. A moment or two later, and the black and bloated face, the choking sounds, and the absolute suspension of all respiratory movements, show very plainly that the formation of red blood is arrested for the time. During the whole course of the convulsion, indeed, the state is one of suffocation. During the convulsion, that is to say, the supply of arterial blood is cut off at the fountain-head.

There is, however, one fact which, at first glance, might seem to show that there is an increased injection of arterial blood during the convulsion. Such injection is manifestly very imperfect at the onset of the fit, for upon no other supposition can we explain the corpse-like paleness of the countenance, and the feeble and imperceptible pulse. But if the finger be kept upon the wrist during the convulsion, it will be found that the pulse will go on rising until it has acquired a force and fulness which it never had in the interparoxysmal period ; and if the hand be placed on the breast, it will be found also that this rising of the pulse is accompanied by increased action of the heart. These facts are evident and unmistakable, but they do not show, as without reflection they might seem to do, and as they are often supposed to do, that *red blood* is being injected in greater quantity into the arteries during the convulsion.

When the process of respiration is arrested, the right side of the heart and the venous system generally are soon gorged and distended with black blood. Under these circumstances, indeed, the gorged and distended state of the right side of the heart may reach a point in which the folds of the tricuspid valve are forced widely apart, and an opening left, through which the beatings of the ventricle are made to tell almost as much in driving the blood back through the auricle into the veins, as in sending it onward through the pulmonary artery into the lungs. But it is not right to suppose that the left side of the heart and the arterial trunks are empty of blood ; and this may be readily verified by watching the changes which take place in the carotid and jugulars of a rabbit during the process of suffocation. On exposing the vessels, the artery is seen to be filled with red and the veins with black blood. On suffocating the animal by tying a ligature around its windpipe, the color of the blood in the artery darkens rapidly, and in about two minutes and a half it is every whit as black as that of the blood moving in the neighboring vein. Nor is the vein gorged and distended and the artery comparatively empty. On the contrary, the artery is felt to pulsate as strongly under the rush of black blood as it did previously under the rush of red blood. Nay, *the pulse of the black blood is actually stronger than the pulse*

*of the red blood;* for, on testing with the hæmadynometer, the late Professor John Reid (who first directed attention to these facts, and who has investigated the condition of the circulation in asphyxia more carefully than any other observer) found the mercury highest at the moment when the blood in the artery had become thoroughly venous and black.

A full pulse and a throbbing heart, therefore, must be looked upon as natural accompaniments of asphyxia; and thus the full pulse and the throbbing heart of the epileptic paroxysm, instead of showing that a large quantity of *red blood* is being injected into the arteries at the time, may show that these vessels are then laboring under a load of *black blood*, as they do in asphyxia. And that the full pulse and the throbbing heart of the epileptic paroxysm must have this latter significance is evident, for the livid, black, and bloated head and neck, and the complete suspension of all respiratory changes, show very clearly that black and not red blood is coursing through the vessels at this time.

When the convulsion is over there is little to notice in the state of the circulation and respiration. When the spasms cease, the respiration is speedily re-established, and the re-admission of arterial blood into the system may be attended with some transient and inconsiderable febrile reaction ; but this reaction has nothing to do with the convulsion, for when reaction is present, convulsion is absent; and if convulsion returns, it is not till every trace of reaction has first taken its departure.

Regarding, therefore, these facts—the corpse-like paleness and the comparative pulselessness at the onset of the paroxysm, and the signs of positive and unequivocal suffocation by which this stage of paleness and pulselessness is succeeded—and remembering the previous arguments, which show that the convulsion is not to be ascribed to the presence of the stimulus of venous blood, there appears to be only one conclusion, and this is, that the convulsion of epilepsy is connected with the want of a due supply of arterial blood in the vessels.

Nor is it an objection to this view that the convulsions cease when the blood has become thoroughly deprived of its arterial properties. In order to discharge their office of conductors, it is certain that the nerves must be supplied with a sufficient quantity of arterial blood. If, for example, the principal vessel of a limb be tied, the nerves of that limb, wanting their due supply of blood, are unable to carry messages to the mind, or to transmit mandates from the mind to the muscles, until the collateral circulation is sufficiently established; and hence it is a fair inference that there must be a point in the process of suffocation where, wanting a due supply of arterial blood, the nerves must cease to be conductors, and where, consequently, the convulsions will come to an end; for upon any hypothesis, the convulsions will come to an end when the nervous centres cease to be in proper connexion with the muscles.

But, it may be asked, is there no change in the blood itself? Is there not some important truth in the "humoral theory of epilepsy," as recently advanced in this place by the late lamented Dr. Todd? "I hold," said this distinguished physician, "that the peculiar features of an epileptic seizure are due to the gradual accumulation of a morbid material in the blood, until it reaches such an amount that it operates upon the brain in, as it were, an explosive manner; in other words, the influence of this morbid matter, when in sufficient quantity, excites a highly polarised state of the brain, or of certain parts of it, and these discharge their nervous power upon certain other parts of the cerebro-spinal centre, in such a way as to give rise to the phenomena of a fit. A very analogous effect is that which results from the administration of strychnia, which is best seen in a cold-blooded animal, like the frog. You may administer the drug in very minute quantities for some time without producing any sensible effect; but when the poison has accumulated in the system up to a certain point, then the smallest increase of dose will immediately give rise to the peculiar convulsive phenomena. This is the humoral theory of epilepsy. It assumes that the essential derangement of health consists in the generation of a morbid matter, which infects the blood; and it supposes that this morbid matter has a special affinity for the brain, or for certain parts of it, as the strychnia, in the case just cited, exercises a special affinity for the spinal cord. The source of this morbid matter is probably in the nervous system,—it may be in the brain itself. It may owe its origin to a disturbed nutrition—an imperfect secondary assimilation of that organ—and in its turn will create additional disturbance in the functions and nutrition of the brain." And again: "According to the humoral theory, the variety in the nature and severity of the fits depends on the quantity of the poisonous or morbid material, and on the part of the brain which it chiefly or primarily affects. If it affect primarily the hemispheres, and spends itself, as it were, on them alone, you have only the epileptic vertigo. If it affect primarily the region of the quadrigeminal bodies, or if the affection of the hemispheres extend to that region, then you have the epileptic fit fully developed."

This theory is based upon the well-known connection between the presence of urea in the blood, or carbonate of ammonia resulting from the decomposition of urea—the result of defective renal action—and one form of epilepti-form convulsion; and it might also have been based upon the connection between convulsion and blood overloaded with bile. But if there is any evidence in these facts in favor of the existence of this hypothetical morbid material, there is none in favor of the idea that the *modus operandi* of the material is in exciting a highly polarized state of the brain, if by this state is meant anything like a condition of excitement. On the contrary, it is certain (as will be shown in the next lecture) that the action of the brain and of the nervous system generally is reduced to the very lowest ebb at the time when convulsion is brought about by the accumulation of urea and bile in the blood; and it is not less certain that strychnia, instead of acting as Dr. Todd supposes it to act—that is, by exciting a highly polarized state of certain parts of the nervous centres, acts by reducing the stimulating powers of the blood, and by diminishing the electrical action of both nerve and muscle.

There is little doubt, however, that retained excretions must play an important part in the production of epilepsy. A free discharge in the office of excretion, not only in the kidneys and liver, but in every excretory organ, is essential to the preservation of healthy blood; and it may well be believed that an imperfect discharge of the office of excretion, *in one or other of the excretory organs*, may lead to the accumulation of effete matter may be a not unimportant cause in bringing about an attack of epilepsy. But there is no reason for supposing that the blood under these circumstances becomes more stimulating. On the contrary, the conclusion which arises out of the history of the cases where the urine or bile is suppressed, is the natural conclusion, and this is, that blood thus altered is less fit to discharge its several offices; in other words, less stimulating.

Nor does there appear to be any reason for supposing that venous congestion has a more important part to play in the production of epilepsy than that which has been assigned to arterial injection. No doubt the veins of the head and brain generally are congested from a very early moment, but there is a moment antecedent to this in which the death-like pallor of the face is a sufficient proof that the veins were emptier than usual before they became congested. At any rate, the acknowledged anatomical difficulty must be overcome before it can be supposed that Dr. Marshall Hall's hypothesis of *trachelismus*—or the prevention of the return of blood from the brain by the spasm of certain muscles in the neck—has anything to do with the causation of epilepsy.

It would seem, then, as if there was something utterly uncongenial between epilepsy and arterial excitement. It would seem, indeed, as if the spasms, as well as the loss of consciousness and sensibility, were connected with want of arterial blood—empty vessels in the first instance, vessels filled with black blood afterwards. It is not improbable, also, that the blood may have been previously rendered less stimulating by the retention of something which ought to have been eliminated by one or other of the organs of excretion. In a word, the phenomena are entirely in harmony with the previous considerations respecting muscular motion; for according to them, the action of arterial blood is to antagonize contraction, and not cause it.

Interrogating the nervous system, the facts are found to have that theoretical significance which the state of the circulation and respiration would lead us to expect.

These facts will scarcely warrant the idea that epilepsy is connected with anything approaching to over-action of the *brain proper*. On the contrary, everything seems to point to a state which is the very opposite of over-activity. Thus, the comparative want of memory, intelligence, fancy, and purpose, which marks the interparoxysmal condition; the utter annihilation of everything mental in the fit itself; and the gloom and prostration following the fit, are facts which can have no double meaning.

Nor is a contrary opinion to be drawn from the morbid appearances which are disclosed after death. If these chance to indicate previous inflammation, it does not follow that convulsion had any direct connection with the inflammation as inflammation; on the contrary, the convulsion may have happened before or after the inflammation, when the energies of the brain were prostrate or exhausted—an alternative which we shall see to be the correct one when we come to speak of epileptiform disease connected with special disease of the brain. And surely it is not possible to draw any but one conclusion from the appearances which are common to epilepsy and dementia—pallor of the grey substance, atrophy, chronic softening and induration, dropsical effusion, and the rest?

But what of the state of the *medulla oblongata*? For, as Professor Schrœder Van der Kolk has well shown, the seat of the characteristic spasms, the bilateral character of the spasms, and the appearances presented after death, all point to this organ as one which is specially concerned in bringing about the epileptic paroxysm.

The spasms of epilepsy begin in muscles which receive nerves from the medulla oblongata—in muscles, that is to say, which are supplied by the facial, the accessory, the hypoglossal, and the portio minor trigemini; in slighter cases they are limited to these muscles. The spasms of the walls of the chest and abdomen, which are the most prominent and marked features in the complete attack of epilepsy, and which may be so fierce and unyielding as to cause fatal suffocation, also point to the same nervous centre; for a similar state of things is brought about by the action of a strong stimulus upon the great afferent nerve of this centre—the pneumogastric.

The bilateral character of the spasms is another argument that the medulla oblongata is especially affected in epilepsy. The lateral halves of this organ are connected in the most intimate manner by transverse fibres and commissures—much more intimately than the lateral halves of the brain and spinal cord; and hence it is that the corresponding nerves belonging to the two sides of the medulla oblongata are under a stronger physical necessity to act together than that which rules the corresponding nerves belonging to the two sides of the brain and spinal cord. In the case of the two latter centres, the nerves belonging to one side may be paralysed or otherwise affected, without any obvious injury to the nerves of the other side; but not so in the case of the latter centre. Indeed, it is evident that the actions which emanate from the latter centre—the play of the features, the motion of the tongue, the vocal adjustments of the larynx, the respiratory movements, &c.,—must at once come to an end unless there be the strictest sympathy and concert in the action of the corresponding nerves of the two sides. Now, in epilepsy the spasms are always more or less *bilateral*, and for this reason, therefore it may be supposed that they have some special connexion with a nervous centre of which one lateral half cannot act without the other.

The appearances after death point also to the medulla oblongata as especially concerned in the production of epilepsy. In an early stage of the disorder, we may fail to find any characteristic changes; but, in confirmed cases, the texture is harder than natural, from the interstitial deposit of a minutely-granular albuminous matter, or else softened, swollen, and exhibiting signs of evident fatty degeneration. The posterior half of the medulla oblongata is redder and more hyperæmic than it ought to be; and, on examining the blood vessels in this congested portion, they are found to be of thrice their natural dimensions, and with their walls much thickened and altered—this dilatation and alteration being chiefly in the corpus olivare and in the course of the hypoglossus in the case of epileptics who bite their tongue, and in the course of the roots of the vagus in the case of epileptics who do not bite their tongue.

It is evident, then, that the medulla oblongata is especially affected in epilepsy; but it does not follow, as Professor Van der Kolk supposes, that the essential cause of the convulsive affection is to be found in an exalted sensibility and activity of the ganglionic cells of this centre.

In favor of this view,—that epilepsy is dependent upon exalted sensibility and activity of the ganglionic cells,—appeal has been made to the fact of spasm, to the presence of a full, bounding pulse, and to the freedom from attack which is for some time the fruit of an attack, particularly if this has been violent; but the answer is not necessarily that which Professor Van der Kolk supposes it to be. After what has been said about muscular motion in the first lecture, it is not possible to allow that spasm in itself is an argument in favor of exalted sensibility and activity in ganglionic cells. After what has just been said about the phenomena of the circulation in epilepsy, it is impossible to allow that the condition of the circulation favors spasm by bringing about a more active state of the medulla oblongata, (the functional activity of this, as of every other organ, being in direct proportion to the activity of the circulation of *red* blood in the organ;) for it has been seen that the bounding pulse, to which reference is made, is filled with *black* blood, and not with red. Nor can the freedom from attack, which is for some time the fruit of an attack, be appealed to as a certain proof that the attack is the sign of the discharge of some overcharge of excitability previously present. On the contrary, it may be argued with some degree of plausibility, from certain facts which have to be mentioned in the next lecture, that the attack was preceded by depression of the circulation and inervation, that the convulsion supervened when this depression had reached a certain point, and that the recurrence of the attack was prevented for a time by the state of reaction in the circulation and inervation, which is a consequence of the convulsion. The case may be one, indeed, of which the history of the rigors of ague may serve as no inapt illustration; for here we have first, the circulation failing more and more until the pathos of the cold stage is reached; and secondly, a state of reaction which banishes the rigors most effectually so long as it continues.

It would even seem as if appeal might be made to the appearances after death, and to the actual condition of the circulation in the fit, for positive arguments against the idea of anything approaching to exalted action of the medulla oblongata.

The signs of fatty degeneration can have but one significance—under-action, not over-action. The interstitial deposit, also implies an equivalent absence of healthy nerve-structure, and so does the dilated condition of the blood vessels; and this absence of nerve-structure must necessitate a corresponding absence of nervous action. The appearances after death, indeed, if they show anything, show that the medulla oblongata of the epileptic is *damaged in structure*, and because damaged in structure, *weaker in action* than it ought to be.

The great argument against the idea of anything like over action of the medulla oblongata in epilepsy, however, is to be found in the state of the circulation; for if, as may safely be assumed, the activity of any organ is in direct relation to the activity of the circulation of *red blood* in that organ, how far from anything like-overaction must be the state of things in which, as is the case in the epileptic paroxysm, the vessels are at first comparatively empty of red, and afterwards completely filled with black blood.

Nor can the curious discovery of Dr. Brown-Sequard, that certain injuries of the spinal cord are followed by an epileptiform affection, be construed into an argument that there is anything like a state of exalted action of the *spinal cord* in epilepsy. This curious result which is brought about by puncturing or dividing more or less completely almost any part of the spinal cord, developed, not immediately, but in the course of three or four weeks after the injury. The attacks once developed, occur spontaneously at various intervals, often several times a day; they may also be brought on by pinching or otherwise irritating the portion of the skin which corresponds to the region of the whiskers in man.—This excitable spot is supplied by twigs belonging to the suborbitory, the auriculo-temporalis, the second, and perhaps the third cervical nerves; and it is a curious fact, that the irritation which brings on a fit when applied to the skin in which these twigs terminate has no such effect when applied to the twigs themselves. Any other part of the skin may be pinched or irritated with impunity, but this one spot can scarcely be touched without at once bringing on a fit.

These facts are very curious, and in the main, very unintelligible; but this much at least is evident, they do not countenance the idea of any over-action of the spinal cord in epilepsy. The fact that the epileptiform affection does not make its appearance until four or five weeks after the injury, would appear to show very clearly that the fits have nothing to do with that local inflammation in the cord which may be supposed to have been set up in the first instance by the injury. After such a lapse of time, indeed, is it not the natural conclusion, that any over-action of the cord arising from the inflammation produced by the injury must have died out, and left the cord damaged, weakened, under-action? Nor is a contrary conclusion to be drawn from the excitable condition of the nerves proceeding from the neighborhood of the cheek or cheeks; for both sides of the face are thus affected, if both sides of the spinal cord have been injured. What the full significance of this curious fact may be, we may have yet to learn; but at any rate there is no reason to suppose that this excitable condition of the skin implies an over-acting condition of the nerves or nervous centres concerned in the phenomenon. The excitable portion of the skin is not over-sensitive, for the animal manifests no signs of uneasiness when it is handled immediately after a fit. Over-sensitiveness, moreover, would seem to have nothing to do with the matter. At any rate pain, and not convulsion, is the consequence of handling those portions of the skin of the animal which may have been rendered highly hyperæsthetic by the injury to the cord which brought on the convulsions. It is certain also that a somewhat similar condition of excitability is brought on when, as in several experiments related in the first lecture, the skin is cut off from the full influence of the nervous centres; and hence the natural inference would be that the action of the nervous centres in the epileptic guinea-pig is *minus* rather than *plus*.

As in the former instances, however, so here, we turn to the condition of the circulation and respiration in order to know what is the actual functional condition of the spinal cord in epilepsy; and so turning, we see that the action of the cord under these circumstances must be almost or altogether *nil*. For what action can there be when little or no arterial blood is injected into the vessels?

A similar argument will also dispose of the idea of over-activity of the *ganglia of the sympathetic system* as a cause of epilepsy. It is very possible that the contracted state of the arteries, which is implied by the death-like pallor of the countenance and the comparative pulselessness at the wrist, may show that the coats of the vessels are in a state of spasm; and it is also possible that the cause of this spasm may have to be sought in the sympathetic system; but it does not follow that over-action of this system is the cause. On the contrary, the experiments of Drs. Kussmaul and Tenner, show most conclusively that strong epileptiform convulsion is possible when the action of the sympathetic ganglia is entirely suspended by arresting the supply of blood to these organs.

And certainly no opposite conclusion is to be drawn from the vague and undefinable sensations or movements, very varying in character, but all comprehended under the term *aura*—sensations of pain, numbness, tingling, or a feeling of cold vapour; movements of shuddering or spasm, beginning in a distant part and travelling towards the head; for the most probable interpretation of these symptoms is that of Dr. Watson—that they are in some degree analogous to the numb and tingling feelings which are the frequent precursors of paralysis and apoplexy, or to the globus of hysteria—phenomena which by the most perverse process of reasoning can scarcely be supposed to indicate other than a state of defective innervation some where.

But it may be asked, is there nothing else? Is there no peculiar state of the nervous system in epilepsy? Is there no *morbid irritability?* In order to answer this question it is necessary to ask another. What is morbid irritability? It is not inflammation; it is not fever; it is some indefinable and negative state which occurs frequently in teething, in worm disease, in uterine derangement, and in many other cases—a state in which the patient is unusually depressed by depressing influences, and unusually excited by exciting influences. But what is this state? Is it anything more than mere exhaustion? In difficult teething the strength is worn away by pain and want of sleep; in worm disease the parasites help to starve and exhaust the system; in uterine derangement the health is undermined, in all probability, by sanguineous or other discharges. In each case there is unequivocal exhaustion of body and mind, and the signs of morbid irritability appear to be nothing more than the signs of such exhaustion. A weak person is more affected by the several agencies which act upon the body from within and from without, and he is so because he is without some of that innate strength which belongs to the strong person; and the person who is morbidly irritable is in reality, one who, for want of this principle of strength, responds impatiently to the several stimuli, whose office it is to elicit his vital phenomena. In a word, this undue morbid irritability may be nothing else than the natural consequence of that general want of power, the signs of which are written so legibly upon the vascular and nervous system of the epileptic.—There is no necessity then, to look upon this morbid state of irritability as an evidence of the existence of any peculiar condition in some part of the nervous system, for thus interpreted, it only shows that the state of muscular contraction is ill antagonized by nervous influence. Thus interpreted, indeed, morbid irritability only becomes another name for inefficient inervation.

The *theory of simple epilepsy*, therefore, which may be deduced from a consideration of the facts relating to the nervous system is in harmony with that to which we have been led by a review of the state of the circulation and respiration in the epileptic; and this theory is one which tallies as completely with the view of muscular motion set forth in the first lecture, as it disagrees with that commonly received opinion according to which the muscles are supposed to contract convulsively because they are subjected to excessive stimulation. —*Lancet*, *May* 26*th*, 1860, *p.* 512.—*Braithwaite's Retrospect.*

